# Supplementary figures and images for: The association between fetal fraction and pregnancy-related complications among Chinese population
Source: PLoS One. 2022 Jul 12;17(7):e0271219. doi: 10.1371/journal.pone.0271219 (PMC9275705; doi:10.1371/journal.pone.0271219)

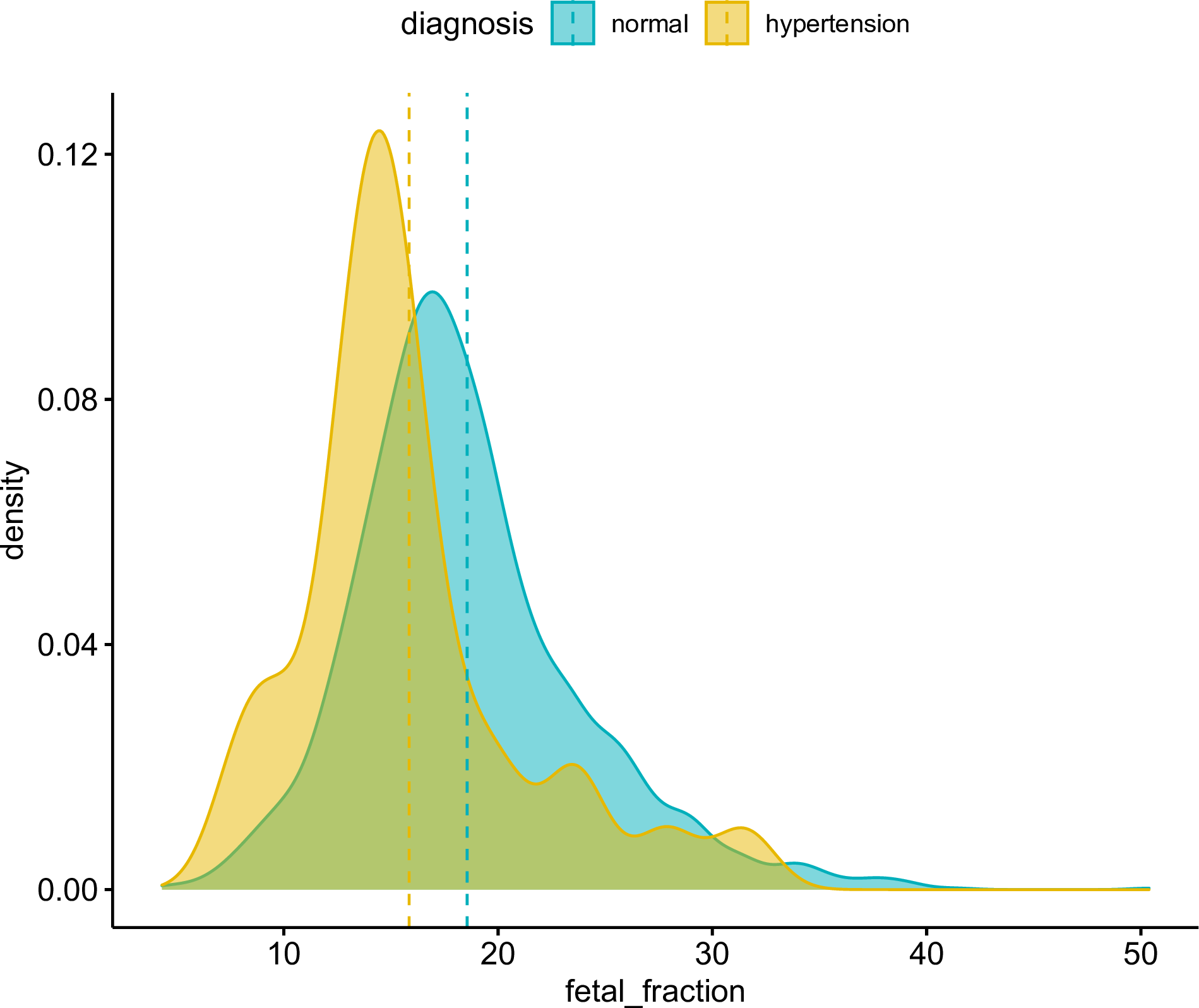

Supplement: S1 Fig — Density plot of fetal fraction from gestational hypertension samples and normal samples in 2 different colors. Dotted lines represent the mean values of fetal fraction from the 2 groups. (TIF) [file pone.0271219.s001.tif]

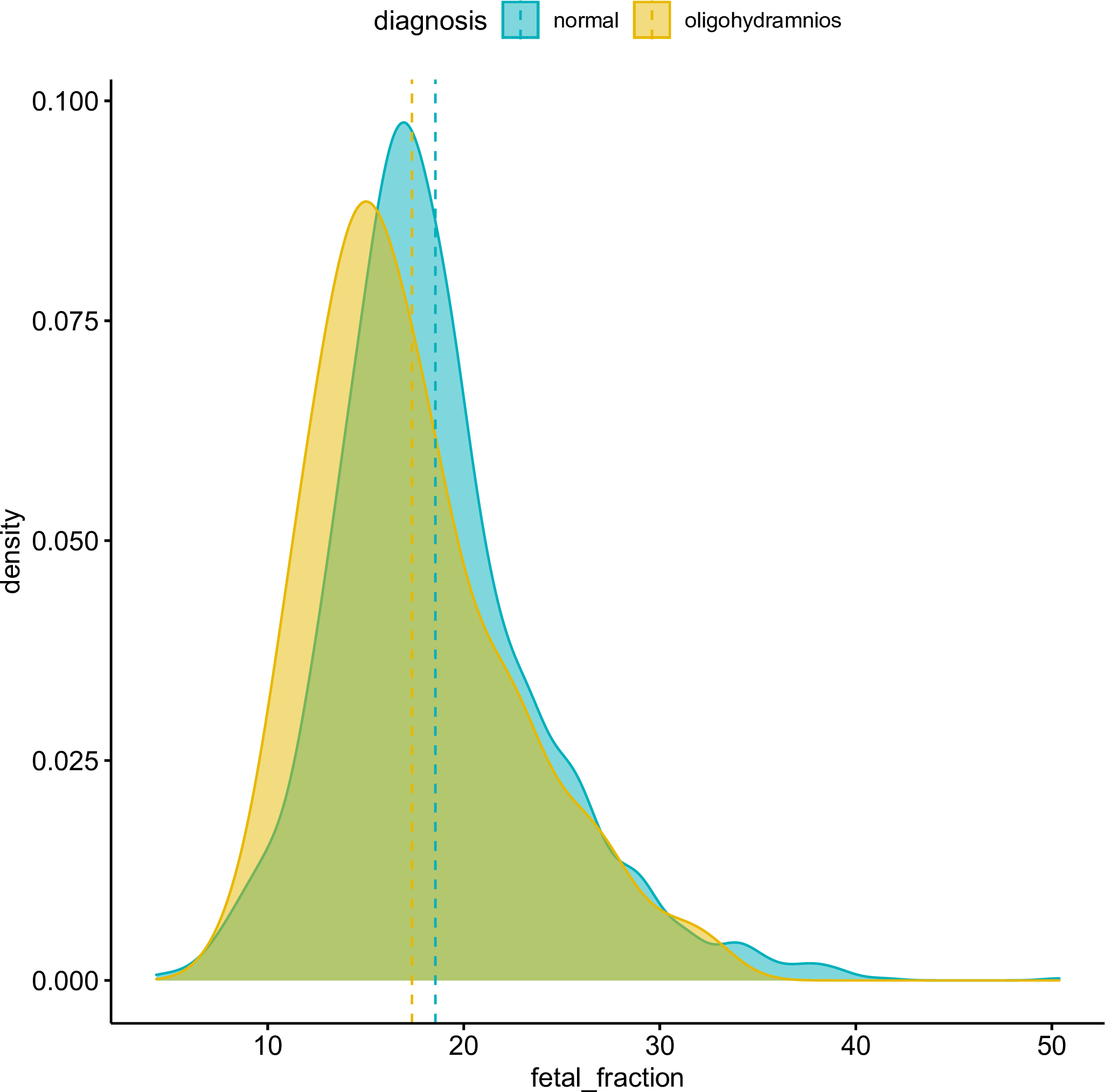

Supplement: S2 Fig — Density plot of fetal fraction from oligohydramnios samples and normal samples in 2 different colors. Dotted lines represent the mean values of fetal fraction from the 2 groups. (TIF) [file pone.0271219.s002.tif]
